# The Role of HCMV and HIV-1 MicroRNAs: Processing, and Mechanisms of Action during Viral Infection

**DOI:** 10.3389/fmicb.2017.00689

**Published:** 2017-04-21

**Authors:** Doriana Fruci, Rossella Rota, Angela Gallo

**Affiliations:** ^1^Immuno-Oncology Laboratory, Oncohaematology Department, Bambino Gesù Children’s Hospital, Istituto di Ricovero e Cura a Carattere ScientificoRome, Italy; ^2^Angiogenesis Laboratory, Oncohaematology Department, Bambino Gesù Children’s Hospital, Istituto di Ricovero e Cura a Carattere ScientificoRome, Italy; ^3^RNA Editing Laboratory, Oncohaematology Department, Bambino Gesù Children’s Hospital, Istituto di Ricovero e Cura a Carattere ScientificoRome, Italy

**Keywords:** microRNA, HCMV, HIV, immune system, cancer

## Abstract

Viruses infect host cells releasing their genome (DNA or RNA) containing all information needed to replicate themselves. The viral genome takes control of the cells and helps the virus to evade the host immune system. Some viruses alter the functions of infected cells without killing them. In some cases infected cells lose control over normal cell proliferation and becomes cancerous. Viruses, such as HCMV and HIV-1, may leave their viral genome in the host cells for a certain period (latency) and begin to replicate when the cells are stressed causing diseases. HCMV and HIV-1 have developed multiple strategies to avoid recognition and elimination by the host’s immune system. These strategies rely on viral products that mimic specific components of the host cells to prevent immune recognition of virally infected cells. In addition to viral proteins, viruses encode short non-coding RNAs (vmiRNAs) that regulate both viral and host cellular transcripts to favor viral infection and actively curtail the host’s antiviral immune response. In this review, we will give an overview of the general functions of microRNAs generated by HCMV and HIV-1, their processing and interaction with the host’s immune system.

## Introduction

MicroRNAs (miRNAs) are short (∼22 nucleotides) single-stranded non-coding RNA molecules that negatively regulate gene expression at post-transcriptional level. The miRNA maturation machinery involves several steps and multiple proteins in both nucleus and cytoplasm. miRNAs can be transcribed as part of independent primary transcripts (pri-miRNAs), which are mainly generated by RNA polymerase II (Lee et al., 2004; Ozsolak et al., 2008), and display all features commonly associated with Pol II-mediated transcription, such as histone marks, CpG islands, transcription factor binding sites (Cai et al., 2004). In the canonical miRNA biogenesis pathway, the Microprocessor complex, a multiprotein complex with Drosha and Di George Syndrome critical region gene 8 (DGCR8), cleaves the double-stranded pri-miRNA generating a hairpin-shaped RNA molecule (pre-miRNA) of about 70–100 bp (Denli et al., 2004; Han et al., 2006). This process can be modulated by different proteins (Suzuki et al., 2009; Trabucchi et al., 2009). Once generated, nuclear pre-miRNAs are exported to the cytoplasm by the exportin-5/Ran-GTP complex to be further processed by the RNase III enzyme Dicer (Grishok et al., 2001). The pre-miRNA processing is finely regulated and its inhibition affects miRNA-mediated differentiation in embryonic stem cells, embryonal carcinoma cells and certain primary tumors. An example is provided by Lin-28, an developing regulated RNA binding protein, which promotes cell proliferation and tumorigenesis of embryonic cells by affecting let-7 maturation (Viswanathan et al., 2008). The activity of Dicer can be either enhancing or inhibiting by A-to-I RNA editing mediated by ADARs enzymes which convert Adenosine to Inosine (Kawahara et al., 2008; Nishikura, 2010; Tomaselli et al., 2013). ADAR1 has been shown to directly bind Dicer, thus increasing the maximum rate of pre-microRNA cleavage by Dicer (Ota et al., 2013). In human adult brain, it has been estimated that approximately 20% of pri-miRNAs can undergo A-to-I RNA editing (Nishikura, 2010; Tomaselli et al., 2013) by affecting miRNAs maturation at different steps (Luciano et al., 2004; Chawla and Sokol, 2014; Tomaselli et al., 2015).

The result of Dicer cleavage is the formation of a double stranded RNA of 22 nt in length whose strand with the less stability is normally chosen as guide strand and transferred to the RNA-induced silencing complex (RISC) for the annealing of miRNAs to the target mRNA, whereas the other strand [the star (^∗^)-strand] is usually degraded. The RISC complex contains Dicer and many associated proteins such as Argonaute (Ago) protein family and the RNA-binding protein TRBP [human immunodeficiency virus transactivating response RNA (TAR) binding protein] (Chendrimada et al., 2005).

Mature miRNAs recognize their target mRNAs through 6–8 nucleotides (the *seed* region) at the 5′ end of the miRNA. Complete complementarity between the miRNA and target mRNA sequence directs mRNA degradation, while absent of perfect complementarity will silence the gene target by preventing its translation (Lim et al., 2005). A given miRNA may have hundreds of different mRNA targets, and a given target mRNA might be regulated by several miRNAs. These evidences suggest that the biogenesis of miRNAs is extremely complex and regulated at different levels, thus highlighting the importance of these short RNA molecules in crucial cell programs including viral infection.

**FIGURE 1:**
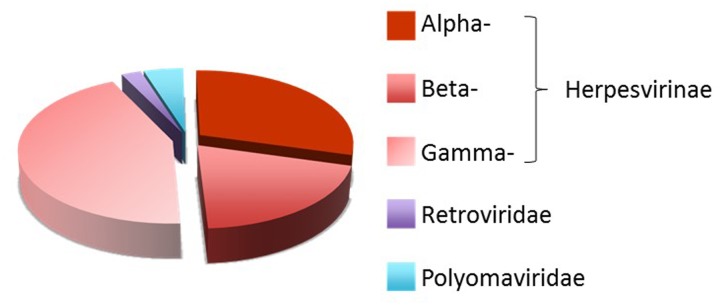
**Distribution of human viral miRNAs.** Distribution of human viral-encoded miRNAs listed in the miRBase collection (http://www.mirbase.org/) (Kozomara and Griffiths-Jones, 2014).

Viruses have developed multiple strategies to avoid recognition and elimination by the host’s immune system. These strategies rely on viral products that mimic specific components of the host cells to prevent immune recognition of virally infected cells. In addition to viral proteins, viruses encode miRNAs (vmiRNA) that regulate both viral and host cellular transcripts during viral infection. The first vmiRNAs was identified in a cell line latently infected with Epstein–Barr virus (EBV) (Pfeffer et al., 2004), a member of the *Herpesviridae*, that in humans is associated with Burkitt’s lymphoma, Hodgkin’s disease and nasopharyngeal carcinoma (Raab-Traub, 2007). Currently, different members of *Herpesviridae, Polyomaviridae* and *Adenoviridae* families are known to express vmiRNAs (Skalsky and Cullen, 2010). To date, 172 viral-encoded mature miRNAs are listed in the miRBase collection^1^ (Kozomara and Griffiths-Jones, 2014), the majority of which (93%) belongs to the three subfamilies (*Alphaherpesvirinae, Betaherpesvirinae* and *Gammaherpesvirinae*) of *Herpesviridae* family (**Figure 1**).

Biogenesis of vmiRNAs largely relies on host-derived machineries. The common pathway of vmiRNA maturation involves the transcription from host RNA polymerase II into a long transcript precursor known as viral pri-miRNA (vpri-miRNAs), which is trimmed by the host RNase III endonuclease Drosha microprocessor into approximately 80 nt long hairpin structures, known as viral pre-miRNAs (vpre-miRNAs). Vpre-miRNAs are rapidly exported to the cytoplasm where a specialized multi-domain ribonuclease III enzyme, known as Dicer, removes the loop structure leaving the vmiRNA duplex. Some functional vmiRNAs, such as murine γ-herpesvirus 68 (MHV68) miRNAs, are produced by host RNA polymerase III and tRNase Z, independently from the microprocessor-Drosha component (Bogerd et al., 2010), whereas others are generated by different additional non-canonical pathways (Pfeffer et al., 2005; Diebel et al., 2010, 2014; Kincaid et al., 2012; Burke et al., 2014; Feldman et al., 2014; Kincaid et al., 2014; Whisnant et al., 2014).

In general, virus and the host-cell’s miRNAs use different mechanisms to interfere each other. Viruses can either block or impair the host cells miRNA pathway by interacting with key proteins (Lu and Cullen, 2004; Bennasser et al., 2006), synthesize and regulate their own miRNAs (Grundhoff and Sullivan, 2011; Harwig et al., 2014), exploit cellular miRNAs to complete their replication cycle (Luna et al., 2015). Conversely, host cells can target vmiRNAs with endogenous miRNAs (Lecellier et al., 2005; Delorme-Axford et al., 2013; Bai and Nicot, 2015). The complex interplay between viruses and host cells usually favors viral infection by either reducing immune recognition or promoting cell growth and lytic or latent infection. One of the best examples of virus-host-miRNA interplay important for human infection and diseases come from HCMV. In this review, we summarize the general functions of miRNAs generated by HCMV and their interactions with immune system and we discuss new finding regarding miRNA from HIV-1.

## HCMV MicroRNAs

HCMV is a ubiquitous and highly specific herpesvirus that establishes lifelong latent infections, coexisting asymptomatically with its host in a healthy immune system, with periodic and spontaneous phases of reactivation, lytic replication and virus shedding (Stern-Ginossar et al., 2012). In immunocompromised individuals, such as transplant recipients, HIV-infected patients and individuals with an immunological immaturity, as the fetus *in utero*, HCMV can cause different clinical syndromes the severity of which depends on the degree of immunosuppression (Varani and Landini, 2011). Of note, during pregnancy HCMV primary infections can lead to mental retardation and severe neonatal pathologies.

To persist indefinitely within the host, HCMV has elaborated several strategies that act to subvert host cellular immune responses (Noriega et al., 2012). Many HCMV proteins and vmiRNAs are known to target cellular and viral transcripts to establish and maintain latency (Murphy et al., 2008). The complex interplay between virus and host is further enhanced by post-transcriptional events, such as RNA editing. Indeed, during HCMV infection, the expression of the short form of the RNA editing enzyme ADAR1 (ADAR1-p110) is enhanced and the host miR-376a precursor undergo editing at specific sites. The increased edited-miR-376a during HCMV infection, downregulates HLA-E transcript leading the infected cells more visible by NK cells (Nachmani et al., 2014).

**FIGURE 2:**
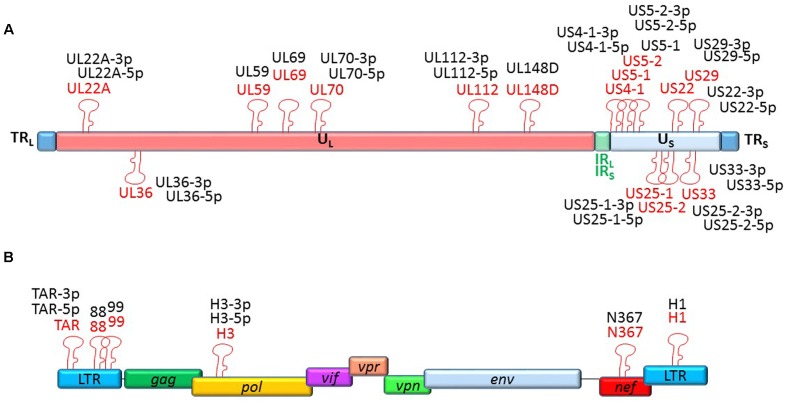
**Genomic organization of the miRNAs encoded by HCMV and HIV-1.** Location of precursors (red) and mature (black) miRNAs is shown by hairpin loop structures along the linear representation of the HCMV **(A)** and HIV-1 **(B)** genomes. miRNAs shown above the line representing viral double-strand DNA are transcribed in sense direction (from right to left), while those shown below are transcribed in the opposite direction.

To date, 15 stem-loop precursors and 26 mature HCMV miRNAs are deposited in the miRBase (**Figure 2**). HCMV miRNAs are differently expressed during latent and lytic infection. Of note, only a subset of vmiRNAs are produced during HCMV latency, most of which originate from the unique long (UL) region of the HCMV genome (Meshesha et al., 2016). Interestingly, during reactivation of the virus from latency all known vmiRNAs, including those that were absent during latency, restored their expression. An alternative expression of the two stands of miR-US29 was detected in the lytic and latent infection. Specifically, miR-US29-5p prevailed during lytic infection, whereas miR-US29-3p dominated viral latency, suggesting the presence of a specific mechanism that regulates expression of the two arms of the vmiRNA hairpins during the viral life cycle (Meshesha et al., 2016). It is possible that expression of vmiRNAs during latency is required to manipulate host-signaling pathways and make the latently infected cell ready for reactivation.

HCMV miRNAs are known to target several cellular genes to evade immune system, control cell cycle and vesicle trafficking (**Table 1**). Since this subject has been recently reviewed by Piedade and Azevedo-Pereira (2016), in this review we will deepen the HCMV miRNAs with proved implications in viral evasion from innate and adaptive immune responses.

**Table 1:** HCMV and HIV-1 microRNAs with potential role in viral infection and pathogenesis.

	Targets	Predicted role	Reference
**CMV-encoded miRNA**			
miR-UL36	UL138^*^	Latent infection	Huang et al., 2013
miR-UL36-5p	ANT3	Cell survival	Guo et al., 2015
miR-UL112	MICB	Immune evasion	Stern-Ginossar et al., 2007
	*IE72 (UL123, IE1), UL112/113, UL120/121*	Viral infection	Grey et al., 2007
	IL-32	Immune evasion	Huang et al., 2013
	type I IFN signaling	Immune evasion	Huang et al., 2015
	VAMP3, RAB5C, RAB11A, SNAP23, CDC42	Vesicle pathway	Hook et al., 2014
	TLR2	Immune evasion	Landais et al., 2015
	IKKα, IKKβ	Immune evasion	Hancock et al., 2017
miR-UL148D	CCL5	Immune evasion	Kim et al., 2012
	IEX-1	Cell survival	Wang et al., 2013
	CDC25B	Latent infection	Pan et al., 2016
	ACVR1B	Immune evasion	Lau et al., 2016
miR-US4	ERAP1	Immune evasion	Kim et al., 2011
	QARS	Cell survival	Shao et al., 2016
miR-US5-1	*US7*	Viral infection	Tirabassi et al., 2011
	VAMP3, RAB5C, RAB11A, SNAP23, CDC42	Vesicle pathway	Hook et al., 2014
	IKKα, IKKβ	Immune evasion	Hancock et al., 2017
miR-US5-2	*US7*	Viral infection	Tirabassi et al., 2011
	VAMP3, RAB5C, RAB11A, SNAP23, CDC42	Vesicle pathway	Hook et al., 2014
miR-US25-1-5p	VAMP3, RAB5C, RAB11A, SNAP23, CDC42	Vesicle pathway	Hook et al., 2014
miR-US25-1	E2, BRCC3^∗^, EID1, MAPRE2, CD147	Cell survival	Grey et al., 2010; Fan et al., 2014
miR-US25-2-3p	*eIF4A1*	Viral infection	Qi et al., 2013
miR-US33-5p	STX3	Vesicle pathway	Guo et al., 2015
**HIV-1-encoded miRNA**			
miR-TAR	*ERCC1, IER3* NPM/B23, Caspase 8, Aiolos, Ikaros	Apoptosis	Klase et al., 2009; Ouellet et al., 2013
miR-88		Immune evasion	Bernard et al., 2014
miR-99		Immune evasion	Bernard et al., 2014
miR-H3	*HIV-1 5′ LTR*	Viral replication	Zhang et al., 2014
miR-N367	*NEF*^§^	Viral replication	Omoto et al., 2004
miR-H1	AATF	Apoptosis	Kaul et al., 2009

^*^ In italic viral targets.

*^§^ Hypothetical target.*

The first host cellular mRNA target reported for a HCMV vmiRNA encodes MICB, a stress-induced ligand for the NK cell activating receptor NKG2D critical for the NK cell killing of virus-infected and tumor cells. VmiR-UL112 specifically binds to MICB-3′ untranslated regions (3′UTR) and downregulates MICB expression during viral infection, therefore, leading to decreased binding of NKG2D and reduced killing by NK cells (Stern-Ginossar et al., 2007). This miRNA-mediated MICB inhibition is not exclusive to HCMV, but conserved in the herpesviruses family. Other viral miRNAs encoded by EBV and HHV-8 (miR-BART2-5p and miR-k12-7, respectively), target MICB to escape NK cell recognition (Nachmani et al., 2009). These vmiRNAs exhibit poor sequence homology with HCMV miR-UL112 and target MICB at different binding sites. HCMV miR-UL112 attenuates NK cell activity also by targeting others transcripts, such as IL-32, type I IFN and the toll-like receptor 2 (TLR2) signaling (Landais et al., 2015).

In addition, the HCMV miR-UL148D, one of the most highly expressed vmiRNAs during latent infection, contribute to immune evasion by directly targeting the chemokine (C-C Motif) ligand 5 (CCL5), a chemokine known to attract immune cells to sites of inflammation and tissue damage (Kim et al., 2012). This downregulation was reverted by treatment with a miR-UL148D-specific inhibitor, supporting the role of this agent as therapeutic tool against HCMV infection. VmiR-UL148D also targets the activating receptor type-1B (ACVR1B) in monocytes, resulting in a reduced secretion of IL-6 (Lau et al., 2016). More recently, vmiR-US5-1 and vmiR-UL112-3p have been shown to play important role in modulating NF-κB signaling at late time of infection by reducing the expression of the IKK complex and induce the release of proinflammatory cytokines (Hancock et al., 2017). The limited or delayed secretion of proinflammatory cytokines is thought to be one of the mechanisms of immune evasion exploited by vmiRNAs to limit the recruitment of immune cells and killing of infected cells.

Finally, the HCMV miR-US4-1 was shown to directly target the endoplasmic reticulum aminopeptidase 1 (ERAP1), a key peptidase that trims peptide precursors to their optimal length to bind MHC class I molecules (Kim et al., 2011). The reduced trimming due to HCMV-specific action resulted in an immuno-evasion of HCMV-infected cells (Kim et al., 2011).

All these mechanisms focus on the possibility to enhance viral replication by hindering viral clearance by NK cells and T cells for the period of time necessary to virus to replicate.

## HIV-1 miRNAs

HIV-1 infection cause progressive CD4^+^ T-cell loss making individuals susceptible to get infections and develop a wide range of immunological abnormalities until oncological complications. Although HIV infections are able to induce vigorous antiviral immune responses, HIV-1 replication is not fully controlled by the innate and adaptive immune system. Like many other viruses, HIV-1 has evolved a number of strategies to evade host immune responses, most notably by using viral accessory proteins and RNA.

Recent reports have demonstrated the existence of HIV-1-derived miRNAs from coding and non-coding regions of the viral genome, which regulate both viral and host gene expression (**Table 1**). Despite so, the presence of HIV-1 derived vmiRNAs has been highly controversial and further studies are necessary to better understand their potential role in viral infection and pathogenesis. The importance of viral and cell host miRNAs in the context of HIV-1 infection was first suggested by silencing of Drosha and Dicer leading to significant enhancement of HIV-1 replication (Triboulet et al., 2007). The first description of HIV-1-derived miRNAs came in 2004 from a group that identified a Nef-derived miRNA, named miR-N367 (Omoto et al., 2004). MiR-N367 reduces HIV-1 transcription by blocking Nef expression and long terminal repeat (LTR) transcription (Omoto et al., 2004; Omoto and Fujii, 2005). The HIV-1 transactivation RNA (TAR), which regulates viral translation, also encodes for a vmiRNA (called TAR-miR-5p and -3p) (Ouellet et al., 2008; Klase et al., 2009). TAR-miR has been shown to downregulate host genes (such as ERCC1 and IER3) important for apoptosis and cell survival, thus giving HIV-1-infected cells a survival advantage by preventing host cell death (Klase et al., 2009). Recent studies demonstrated that additional host substrates, including Caspase 8, Aiolos, Ikaros and Nucleophosmin (NPM)/B23, are modulated by TAR-miR (Ouellet et al., 2013). VmiR-H1, located in the LTR has been reported to downregulate the apoptosis-antagonizing transcription factor (AATF) gene product and act as an antagonist of the anti-apoptotic effect mediated by TAR-miR. Additionally, hiv1-mir-H1 can downregulate the host miR149 expression recognized to target HIV-1 Vpr transcript (Kaul et al., 2009). Recently, Zhang et al. (2014) have reported the existence of a novel HIV-1-encoded vmiRNA called miR-H3 located in the region of the HIV-1 RNA genome that encodes for reverse transcriptase (RT). Overexpression of miR-H3 increases viral production and mutations within miR-H3 sequence significantly impair the viral replication of wild-type HIV-1 viruses by targeting HIV-1 5′-LTR (TATA box) (Zhang et al., 2014). Two additional vmiRNAs (called as vmiR88 and vmiR99) were identified in overlapping regions of HIV LTR from viral infected human macrophages (Bernard et al., 2014). They were able to directly stimulate TNFα release by human macrophages through TLR8 activation by a mechanism that is partially dependent on vmiRNA sequence motifs. Of note, vmiR88 and vmiR99 were detected in sera of HIV infected individuals suggesting their role in stimulating recipient macrophages *in vivo* and contributing to chronic immune activation.

## Future Outlook and Conclusion

vmiRNAs have overall a protecting role for the viral infection. By interfering with host cellular proteins and mechanisms of immune evasion, they represent a potential target for future specific therapies. Studies on the complex interplay between viral miRNAs and host genes has been only recently started and further investigations are required to identify more efficient and less toxic therapeutic strategies. The identification of differentially expressed miRNAs during viral infection (for example in HIV-1 infection) may provide a new approach to control disease progression. Finally, understanding the mechanism of vmiRNA-mediated immune evasion will allow us to develop novel strategies to prevent and cure viral infections. Targeting vmiRNAs with molecules such as antagomirs may represent a novel therapeutic strategy to limit chronic immune activation and the progression of viral infection.

## Author Contributions

DF and AG analyzed the literature, wrote the manuscript, and approved the final version for publication. RR analyzed the literature, revised the manuscript and approved the final version for publication.

## Conflict of Interest Statement

The authors declare that the research was conducted in the absence of any commercial or financial relationships that could be construed as a potential conflict of interest.

## Footnotes

1 http://www.mirbase.org/
